# Assembling a Protein-Protein Interaction Map of the SSU Processome from Existing Datasets

**DOI:** 10.1371/journal.pone.0017701

**Published:** 2011-03-10

**Authors:** Young H. Lim, J. Michael Charette, Susan J. Baserga

**Affiliations:** 1 Department of Molecular Biophysics and Biochemistry, Yale University School of Medicine, New Haven, Connecticut, United States of America; 2 Department of Therapeutic Radiology, Yale University School of Medicine, New Haven, Connecticut, United States of America; 3 Department of Genetics, Yale University School of Medicine, New Haven, Connecticut, United States of America; University of Edinburgh, United Kingdom

## Abstract

**Background:**

The small subunit (SSU) processome is a large ribonucleoprotein complex involved in small ribosomal subunit assembly. It consists of the U3 snoRNA and ∼72 proteins. While most of its components have been identified, the protein-protein interactions (PPIs) among them remain largely unknown, and thus the assembly, architecture and function of the SSU processome remains unclear.

**Methodology:**

We queried PPI databases for SSU processome proteins to quantify the degree to which the three genome-wide high-throughput yeast two-hybrid (HT-Y2H) studies, the genome-wide protein fragment complementation assay (PCA) and the literature-curated (LC) datasets cover the SSU processome interactome.

**Conclusions:**

We find that coverage of the SSU processome PPI network is remarkably sparse. Two of the three HT-Y2H studies each account for four and six PPIs between only six of the 72 proteins, while the third study accounts for as little as one PPI and two proteins. The PCA dataset has the highest coverage among the genome-wide studies with 27 PPIs between 25 proteins. The LC dataset was the most extensive, accounting for 34 proteins and 38 PPIs, many of which were validated by independent methods, thereby further increasing their reliability. When the collected data were merged, we found that at least 70% of the predicted PPIs have yet to be determined and 26 proteins (36%) have no known partners. Since the SSU processome is conserved in all Eukaryotes, we also queried HT-Y2H datasets from six additional model organisms, but only four orthologues and three previously known interologous interactions were found. This provides a starting point for further work on SSU processome assembly, and spotlights the need for a more complete genome-wide Y2H analysis.

## Introduction

Direct, pair-wise (binary), physical protein-protein interactions (PPIs) are the foundation of all biological processes. Efforts to elucidate the interaction network of all proteins within a cell or organism — termed the interactome — has helped identify the architectural and functional blueprint of cellular processes in various model eukaryotic organisms, such as yeast [1]–[5], *Drosophila* [6]–[9], *C. elegans* [10]–[14], *Plasmodium* [15], *Arabidopsis* [16]–[17], mouse [18] and humans [19]–[22]. Mapping PPIs has forwarded our understanding of key biological processes such as the mitotic spindle [23], cell polarity [24], the proteasome [25] and the editosome [26]. Furthermore, it has helped assign roles to proteins of previously unknown function [5] and has increased our understanding of and progress against human diseases [27]–[28].

There are two main methods of observing direct PPIs *in vivo*: the yeast two-hybrid (Y2H) and its many derivatives [29] and more recently, the protein-fragment complementation assay (PCA) [30]. In the Y2H, the interaction of bait and prey fusion proteins within the nucleus reconstitutes a transcription factor that up-regulates the expression of a reporter gene. PCA works similarly to the Y2H but occurs in the cytoplasm and replaces the transcription-reporter system with a reconstituted reporter protein capable of metabolizing a toxic compound.

The PPIs of the yeast *Saccharomyces cerevisiae* have been extensively explored. There are currently three genome-wide high-throughput yeast two-hybrid (HT-Y2H) surveys [1]–[3] and one genome-wide PCA study of the yeast interactome [4]. However, while these large-scale Y2H and PCA screening projects have established proteome-wide protein interaction networks (PINs) for yeast, statistical analysis reveals that their combined datasets account for less than 30% of the entire yeast interactome [3]. Furthermore, there is surprisingly little overlap of PPIs between each of the four aforementioned studies and with the literature-curated (LC) interaction dataset. The LC data, which are derived from small scale Y2H studies (otherwise known as the “community” dataset) displays a narrow focus on a few proteins or an interactome sub-network. Despite recent reports to the contrary [21], [31]–[32], the LC dataset is commonly believed to be of higher quality than the HT-Y2H interactions due to its narrow focus on the PPIs of a few well-characterized proteins [33]–[36]. Furthermore, LC studies often report reciprocal interactions (bidirectional interactions where proteins A and B interact as either bait or prey), recapitulate their results via multiple independent orthogonal methods and integrate their findings with other forms of biochemical and genetic data [37]–[51]. The poor PPI overlap among the large-scale screens and with the LC dataset has led to the suggestion that the current HT-Y2H studies were not done to saturation, and therefore must be missing additional interactions [35]. This may be due to a number of reasons. First, most genome-wide HT-Y2H studies do not include all of the protein-coding genes in the yeast genome. The absence of even a few proteins from HT-Y2H screens can significantly reduce interactome coverage [3]. Also, the enormous scope of genome-wide HT-Y2H screens often necessitates a pooling strategy in which up to 96 or more baits or preys are pooled then tested for interaction. However, when pooled, proteins that are toxic when expressed at high levels may display a dominant negative phenotype and interactions involving weakly expressed proteins may be under-reported [35]. Similarly, certain proteins may be inefficiently imported into the nucleus, the site of the Y2H assay. Furthermore, PPIs that are not physiologically relevant (the so called “biological false-positives”) may be obtained for proteins normally residing in different cellular compartments, expressed at different stages of the cell cycle or in different tissues. These confounding factors are believed to result in pooled HT-Y2H screening strategies being less sensitive than array-based one-by-one screens, while potentially containing a higher number of false positive interactions [35], [52].

We focused on mapping the PPIs of the small subunit (SSU) processome, a very large ribonucleoprotein complex comprised of ∼72 proteins and the U3 small nucleolar RNA (snoRNA). This biochemically well defined complex guides the endonucleolytic processing events at sites A_0_, A_1_ and A_2_ that liberate the mature 18S rRNA from the pre-rRNA transcript [53]–[55]. The SSU processome is also believed to chaperone the folding of the pre-18S rRNA and its assembly with ribosomal proteins into the mature SSU of the ribosome.

The SSU processome was originally identified by tandem affinity purification followed by mass spectrometry (TAP/MS) studies [53]–[54], [56]. Subsequent TAP/MS studies expanded the list of SSU processome protein components and provided some of the first data on the presence of sub-complexes [57]–[59]. In all, nearly 70% of all SSU processome proteins have been identified by TAP/MS studies [53]–[54], [57]–[59], with the remaining proteins being identified by other biochemical or genetic methods. Thus, TAP/MS studies have significantly contributed to our current, nearly complete list of the protein constituents of the SSU processome [53]–[54], [57]–[59]. Typically, SSU processome protein components meet the following criteria: *i*) they reside in the nucleolus, the site of ribosome biogenesis, *ii*) their genetic depletion results in an 18S rRNA processing defect and *iii*) they co-immunoprecipitate the U3 snoRNA and/or another SSU processome protein component. There are currently 46 confirmed SSU processome proteins and 26 potential candidates suggested from partial data (Table S1). Some of these proteins have been categorized into the t-Utp/UtpA, UtpB, UtpC, Mpp10, Rcl1/Bms1 and U3 snoRNP sub-complexes by TAP tag co-complex purifications and small-scale Y2H studies [38]–[39], [46], [50], [57]–[59]. However, the majority of SSU processome proteins remain unassigned to a specific subcomplex due to a lack of interaction data. Some proteins may even be components of subcomplexes yet to be identified (Table S1). Identifying the protein-protein interactions of the SSU processome thus becomes the next step in elucidating its assembly, mechanism of function and regulation in pre-rRNA processing.

Considering the SSU processome's well characterized and nearly complete component list, we sought to generate an up-to-date, comprehensive yeast SSU processome PIN by extracting and pooling protein interaction data from existing datasets. After retrieving both high-throughput and literature-curated binary protein interaction data, an interaction map was drawn using Cytoscape. The result is the most current protein interactome map of the yeast SSU processome to date, from which we identify additional interactions within the subcomplexes and some of the first potential interactions linking the various subcomplexes.

## Materials and Methods

### Mining databases for known PPIs

For each SSU processome component, both IntAct (http://www.ebi.ac.uk/intact/) [60] and BioGRID (http://thebiogrid.org/) [61] databases were queried for protein-protein interaction data. These repositories were chosen because they: *i*) provide downloadable data in a tab delimited format for every queried protein, *ii*) each contain PPIs from a different subset of genome-wide high-throughput studies, *iii*) each include PPIs from a different subset of LC studies, *iv*) pool interaction data from various organism-specific databases and *v*) are updated on a monthly basis to include novel interactions. We downloaded a total of 72 files from both IntAct and BioGRID databases, one for each of the 72 SSU processome proteins, totaling 144 spreadsheets by November 5, 2010. These files contained all known interactors — both binary and co-complex — for the query protein, the experimental method used to detect the interaction and the publication reference.

### Organizing the data

All 144 spreadsheets underwent five editing stages to remove information unnecessary to this study and were streamlined into six columns: Bait, Prey, Experimental System (Y2H, Y2H array, Y2H pooling approach, PCA), Literature Code (Uetz *et al.* [1], Ito *et al.* [2], Yu *et al.* [3], Hazbun *et al.* [5], PCA [4] or LC [37]–[51]), Organism (yeast, *Drosophila* and *C. elegans*) and Reference.

#### Edit Stage 1

Data were sorted by experimental methods; non-Y2H and non-PCA derived PPIs were removed. For IntAct files, deleted examples include “tandem affinity purification” and “inferred by author” methods, and for BioGRID, they include “Affinity Capture-MS”, “Phenotypic Enhancement” and “Synthetic Lethality”. Interactions where neither the bait nor the prey represented the query protein were also removed. The IntAct files also included PPI for non-yeast organisms. These data were extracted and edited separately.

#### Edit Stage 2

Proteins with missing names were labeled with the “Standard Name” [62], and all names were kept congruent between IntAct and BioGRID files. Proteins with multiple aliases were labeled with the name most commonly used in literature (*e.g.*, Sas10 was re-named Utp3 and Sik1was re-named Nop56).

#### Edit Stage 3

Columns with information irrelevant to our study were deleted from both sets of data files. For IntAct, 32 data columns were reduced to five columns: bait ID, prey ID, interaction detection method, source (author) and PubMed ID. We also removed the extra columns from BioGRID, cutting nine columns down to the same five of the IntAct files.

#### Edit Stage 4

The 72 BioGRID and 72 IntAct files were merged into one large spreadsheet and duplicates entries were removed. These included identical interactions with the same experimental method and authors, a consequence of some, but not all interactions being reported in both BioGRID and IntAct. However, duplicate interactions identified via different experimental methods or by different research groups were kept.

#### Edit Stage 5

All interactions involving only one SSU processome component (*i.e.*, interactions between an SSU processome component and a non-SSU processome protein) were removed as a function of the SSU processome protein components having been relatively well catalogued biochemically. A “Literature Code” column was added to separate the data into Uetz *et al.* [1], Ito *et al.* [2], Yu *et al.* [3], Hazbun *et al.* [5], PCA [4] and LC [37]–[51] categories.

Completion of all edit stages resulted in one master spreadsheet containing all the query proteins (bait), their interactors (prey), the experimental system used, the literature code, the source organism and the reference (Table S2).

### Interologues – conserved SSU processome PPIs in other species

All downloaded IntAct files also included protein-protein interactions for *C. elegans*, *D. melanogaster*, *H. sapiens*, *S. pombe*, *P. falciparum* and *M. musculus*. Y2H interactions from organisms other than *S. cerevisiae* (non-yeast) were quarantined during *Edit Stage 1* and underwent the remaining editing stages separately. BioGRID pre-categorizes interactions by organism; PPIs for non-yeast organisms were downloaded separately and edited as described above. In *Edit Stage 5* following the IntAct and BioGRID merge, an “Organism” column was added to the master spreadsheet to enable sorting of yeast and non-yeast data. Protein nomenclature specific to the source organism was queried in Homologene (http://www.ncbi.nlm.nih.gov/sites/homologene) [63] to determine the *S. cerevisiae* homologue. Proteins with available Homologene data were renamed as the *S. cerevisiae* homolog (*e.g.*, *D. melanogaste*r CG13097 renamed Mpp10). BLAST analysis [64] was used to identify the yeast homologues of non-yeast proteins not annotated in Homologene [63]. As with the yeast datasets, only PPIs both involving SSU processome components were kept.

### Visualizing the interactome

We used Cytoscape [65], a bioinformatics software used to visualize molecular interaction networks, to convert the spreadsheet files to interactome maps. Nodes refer to proteins and are labeled with the protein's commonly used name. Edges connect two nodes, illustrating a protein-protein interaction. We distinguished in different colored nodes the various known subcomplexes of the SSU processome (see Table S1; green for the t-Utp/UtpA subcomplex, blue for UtpB, yellow for UtpC, gray for the U3 snoRNP proteins, brown for the Bms1/Rcl1 subcomplex and red for Mpp10 subcomplex) and labeled the proteins unassigned to a subcomplex in pink. The numerous RNA helicases of the SSU processome are depicted as diamonds. Cytoscape maps were generated for the SSU processome protein interactions from the Uetz *et al.* [1], Ito *et al.* [2], Yu *et al.* [3], Hazbun *et al.* [5], Tarassov *et al.* [4] and literature-curated datasets [37]–[51]. An additional Cytoscape map was drawn for the merged dataset and included SSU processome interologues.

### Protein motif and domain identification

The motifs and domains present in the SSU processome proteins were identified using the SCOP Superfamily (http://supfam.org/SUPERFAMILY/index.html) [66], the MIPS Comprehensive Yeast Genome Database (http://mips.helmholtz-muenchen.de/genre/proj/yeast/) [67], Pfam domains (http://pfam.sanger.ac.uk/) [68], PROSITE (http://ca.expasy.org/prosite/) [69], SMART (http://smart.embl-heidelberg.de/) [70] and the Conserved Domain Database at NCBI (http://www.ncbi.nlm.nih.gov/Structure/cdd/wrpsb.cgi) [71].

## Results

### Mining databases for known SSU processome protein-protein interactions

We aimed to assemble a protein-protein interaction map of the yeast SSU processome from existing datasets. Three HT-Y2H studies [1]–[3], one PCA dataset [4] and many small-scale LC studies [37]–[51] were queried for PPIs involving the 72 SSU processome proteins. For each protein, one set of data from BioGRID [61] and one from IntAct [60] were downloaded, totaling 144 spreadsheets for the 72 processome proteins. The files were curated to remove interaction detection methods that were neither Y2H nor PCA, such as TAP-Tag, mass spectrometry and genetic interactions. Furthermore, since the list of protein components of the SSU processome has been well characterized [53]–[54], [56]–[59], and is believed to be nearly complete, we also discarded interactions involving non-SSU processome proteins. Most of the PPIs involving non-SSU processome components were with proteins that are poorly characterized, not nucleolar or with no known role in ribosome biogenesis. While deleting these proteins from our analyses may have resulted in the loss of important interactions or potentially novel SSU processome members, we limited our study to nucleolar proteins involved in ribosome biogenesis or known to co-immunoprecipitate other SSU processome constituents such as the U3 snoRNP.

The spreadsheets for each SSU processome protein were merged into a master file and duplicate entries originating from PPIs listed in both BioGRID and IntAct databases were removed (Table S2). The master spreadsheet was sorted by study (Literature Code) to determine how many of the protein interactions for the 72 SSU processome proteins are attributed to each of the three HT-Y2H studies [1]–[3], the PCA dataset [4] and the small-scale LC studies [37]–[51]. An interactome map was drawn using Cytoscape [65] for each dataset to show the extent of SSU processome coverage per study. Finally, the merged master spreadsheet was converted to a Cytoscape map to illustrate the most up-to-date interactome of the 72 SSU processome proteins.

### Expert curation of protein-protein interaction datasets is often required

We initially explored a variety of different PPI databases, including BioGRID [61], IntAct [60], MIPS Mpact [72], DIP [73], STRING [74] and SPIDer [75]. Our survey found that BioGRID and IntAct contained the most complete and up-to-date PPIs, with the other databases containing non-overlapping subsets of the HT-Y2H, PCA and LC datasets. We did, however, identify a number of problems with both the BioGRID and IntAct datasets. Although BioGRID is continuously updated, some published Y2H interactions have yet to be included in the database (as of January 2011), such as the Y2H interactions of the UtpB subcomplex published by Champion *et al.* [38] in November 2008. Thus, BioGRID does not contain a complete inventory of all currently known PPIs. In some instances, the IntAct database had difficulty filtering and reporting interactions involving only the queried protein due to nomenclature conflicts. For example, a query of the proteins Imp3 (“Interacts with Mpp10 #3”) or Imp4 (“Interacts with Mpp10 #4”) retrieved the appropriate PPIs and erroneous included additional PPIs between Mpp10 and other proteins. Furthermore, a few PPIs from one database were absent in the other, such as the interaction between Utp20 and Sof1 reported by Tarassov *et al.* [4], which is included in the IntAct database, but not found in BioGRID. Thus, assembling an interactome from current datasets without expert curation is likely to result in an incorrect protein-protein interaction map.

### Sparse coverage of SSU processome proteins from the three genome-wide HT-Y2H studies

**Figure 1:**
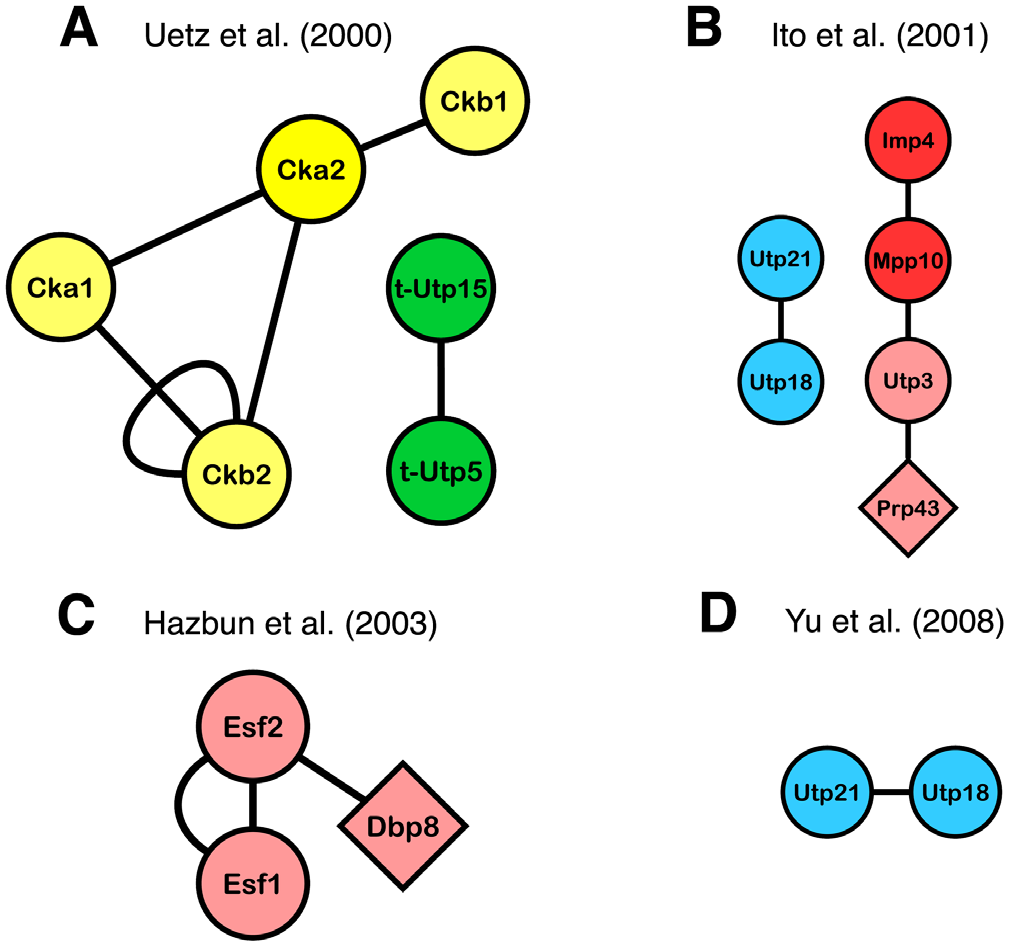
Interaction maps of the SSU processome proteins from existing HT-Y2H datasets. Proteins are colored as described in the Materials and Methods; green nodes refer to proteins of the t-Utp/UtpA subcomplex, blue for UtpB, yellow for UtpC, gray for the U3 snoRNP proteins, brown for Bms1/Rcl1 and red for the Mpp10 subcomplex. Pink nodes refer to proteins that have yet to be assigned to a subcomplex. RNA helicases are depicted as diamonds. Multiple edges, or interactions, linking the proteins represent interactions identified in different studies or reciprocally identified as both bait and prey. Self-interactions are shown as looped edges. **A**) Results from the Uetz *et al.* dataset [1]. **B**) Results from Ito *et al.* dataset [2]. **C**) Results from the Hazbun *et al.* dataset [5]. **D**) Results from the Yu *et al.* dataset [3].

Mining the three genome-wide HT-Y2H datasets for PPIs among SSU processome components revealed disappointingly sparse coverage. The Uetz *et al.* study (2000) [1], which was the first comprehensive HT-Y2H, screened DNA binding domain fusion clones (baits) against both an array and a pool of activation domain fusion clones (preys). For the SSU processome, this yielded five interactions among six of the 72 proteins, as well as one self-interaction for Ckb2 (Fig. 1A and Table 1) [1]. The Ito *et al.* study [2], published in 2001, assembled a yeast interactome by assaying for interactions between the approximately 6,000 proteins of yeast. Sixty-two mating crosses of bait and prey pools were performed with each pool containing 96 different clones as either bait or prey. Their interactions were divided into higher quality “Core” and lower quality “Full” datasets: the former included only the interactions observed 3+ times, while the latter included interactions observed two times. The Ito *et al.* study [2] identified four interactions among six of the 72 SSU processome proteins, all from the lower quality “Full” dataset (Fig. 1B and Table 1). The most recent and third genome-wide HT-Y2H assay, the Yu *et al.* study (October 2008) [3], screened individual baits against pools of 188 different preys. Their dataset revealed only one PPI between two of the 72 SSU processome proteins, Utp18 and Utp21 (Fig. 1C and Table 1). This interaction had previously been identified in the Ito *et al.* dataset (Fig. 1B) [2]. Thus, among the three HT-Y2H datasets, the Uetz *et al.* [1] and Ito *et al.* [2] studies provide the highest coverage of PPIs for SSU processome proteins (Fig. 1A, B, C and Table 1). In all, the three genome-wide HT-Y2H studies account for interactions among only 12 of the 72 SSU processome components (16.7%) and show minimal overlap with the exception of the Utp18-Utp21 interaction reported by Ito *et al.* [2] and Yu *et al.* [3].

**Table 1:** Number of SSU processome proteins (nodes) and the interactions between them (edges) identified in the HT-Y2H, PCA and LC datasets.

Screen	# of nodes	% of nodes	# of edges	% of predicted edges
Uetz *et al.* [1] (Y2H)	6	8.3	6	2.8
Ito *et al.* [2] (Y2H)	6	8.3	4	1.9
Yu *et al.* [3] (Y2H)	2	2.8	1	0.5
Hazbun *et al.* [5] (Y2H)	3	4.2	2	2.7
Tarassov *et al.* [4] (PCA)	25	34.7	27	12.5
Literature-curated [37]–[51] (Y2H)	34	47.2	44	20.4
All merged (not including standalone proteins)	46	63.9	67	31.0
Predicted total	72	100	216	100

Redundant edges were not counted twice. Self-interactions, shown as looped edges in Figs. 1 to [Fig pone-0017701-g002] [Fig pone-0017701-g003] 4, were included in the tabulation. The predicted total number of edges is derived by estimating 3 interactions per protein [3] for each of the 72 SSU processome proteins (72×3 = 216).

A systems biology study by Hazbun *et al.* (2003) [5] used the Y2H methodology to help assign roles to yeast proteins of unknown function. This study individually screened each of 100 essential ORFs of unknown function as baits against an array of approximately 6,000 prey ORFs. From this dataset, we identified three of the 72 SSU processome proteins and two PPIs among them (Fig. 1D and Table 1), with no data overlap with any of the three HT-Y2H studies.

### The genome-wide PCA study contains the best coverage of SSU processome PPIs

**Figure 2 pone-0017701-g002:**
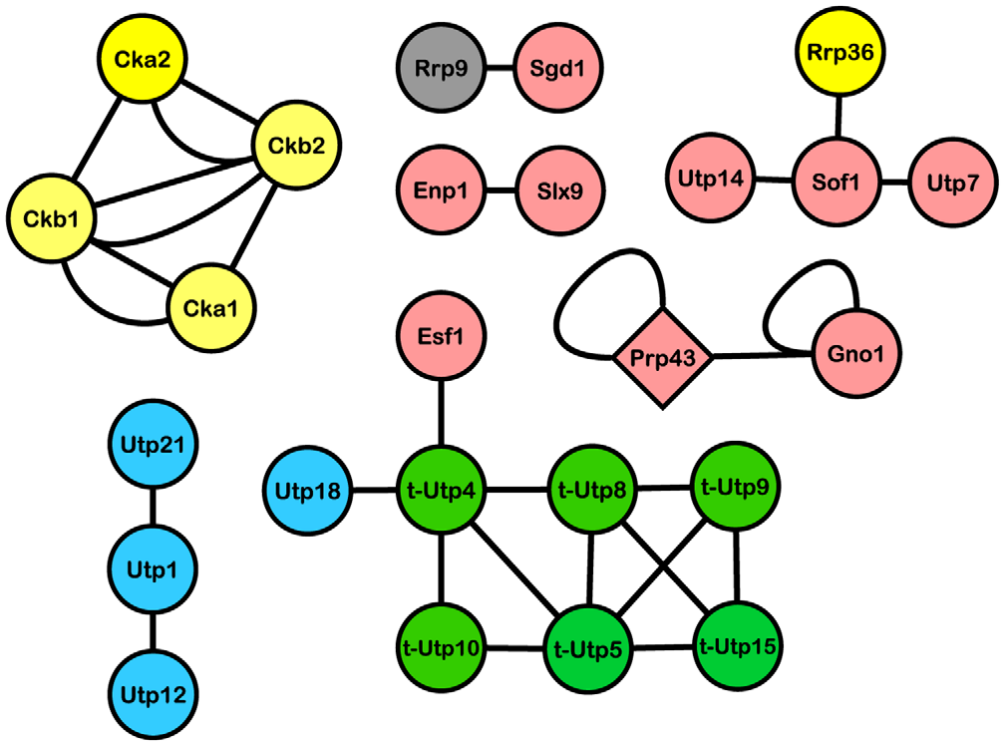
Interaction map of the SSU processome proteins from the PCA dataset. Nodes are colored as in Fig. 1.

The protein fragment complementation assay is an alternative method for identifying direct, physical PPIs. This strategy was used by Tarassov *et al.* in 2008 [4] to compile a forth genome-wide yeast interactome. Unlike the three HT-Y2H studies, the PCA dataset was derived from individual one-by-one matings between haploid yeast strains each carrying bait and prey ORFs. The PCA dataset accounts for 25 of the 72 SSU processome proteins and 27 interactions among them — the highest coverage among the genome-wide studies (Fig. 2 and Table 1) and shows some overlap of PPIs with the Uetz *et al.* [1] dataset.

### The literature-curated dataset contains the best SSU processome coverage overall

**Figure 3 pone-0017701-g003:**
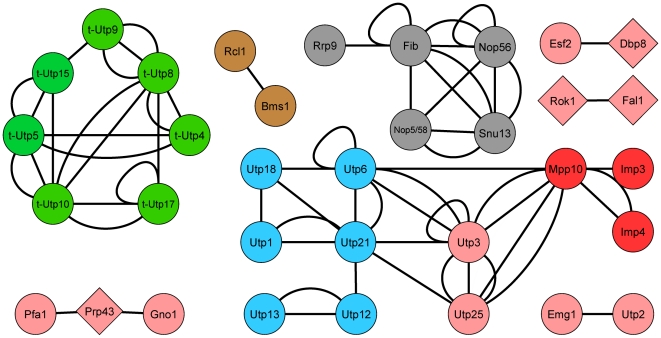
Interaction map of the SSU processome proteins from the LC dataset. Nodes are depicted as in Fig. 1.

The SSU processome protein coverage of the aforementioned datasets was compared to coverage from literature-curated (LC) sources [37]–[51]. These small-scale interaction studies cooperatively account for more SSU processome proteins than any of the individual high-throughput genome-wide datasets [1]–[4]. In all, the LC dataset accounts for 34 of the 72 proteins and 44 interactions (Fig. 3 and Table 1) and displays some overlap with the HT-Y2H [1]–[3] and PCA studies [4].

### Mining for SSU processome interologues

Conserved protein-protein interactions – or interologues – found in multiple organisms, as well as PPIs replicated by multiple studies or distinct experimental methods, carry a higher confidence value and are more likely to represent true interactions [76]–[77]. To determine which interactions have been identified in other organisms, we extracted PPI data for the 72 SSU processome proteins from BioGRID and IntAct for *C. elegans*, *D. melanogaster*, *H. sapiens*, *S. pombe*, *P. falciparum* and *M. musculus*.

**Figure 4:**
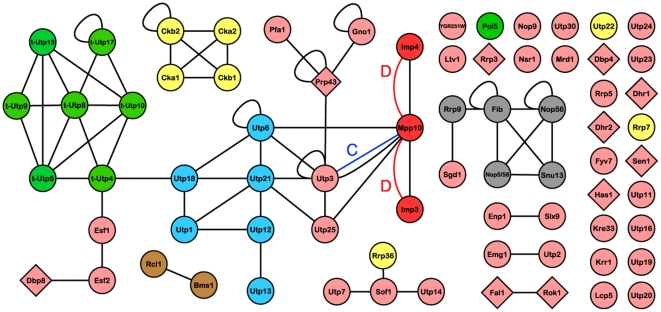
The current, merged SSU processome interactome map from the three HT-Y2H, PCA, LC and interologue datasets. Interologues identified in *Drosophila* (D) [6] and *C. elegans* (C) [10] are also shown, with red and blue edges, respectively. The PPI redundancy (same interactions identified by different studies, methods or reciprocally) was removed from the figure to highlight the interacting partners. Nodes are depicted as in Fig. 1. Standalone nodes depict proteins without interaction data from any of the compiled datasets.

The Cytoscape map of the interologue dataset disappointingly showed only two interactions between Mpp10 and Imp3, and Mpp10 and Imp4 orthologues in *D. melanogaster* [6] and one interaction between Mpp10 and Utp3 orthologues in *C. elegans* (Fig. 4) [10]. These interactions overlap completely with the yeast dataset, thereby further increasing their likelihood. No interactions within the components of the SSU processome were identified in *S. pombe*, *Plasmodium*, human and mouse PPI datasets.

### The first partial protein interaction map of the SSU processome

Merging all the collected yeast and non-yeast PPI datasets [1]–[6], [10], [37]–[51] for the 72 SSU processome proteins provides the first partial protein interaction map of the SSU processome. The Cytoscape map of the merged dataset includes 67 distinct edges, corresponding to 67 different interaction pairs among the 72 queried SSU processome proteins (Fig. 4, Table 1 and S2). Twenty-six out of the 72 proteins (36.1%) did not have any known interacting partners. The LC data (Fig. 3) contributed the largest number of interactions of any dataset (47.2% coverage of the 72 queried nodes and 65.7% of the 67 known edges) followed by the PCA data (34.7% of the 72 nodes, 40.3% of the 67 known edges). The other studies each account for less than 10% of the 67 currently known PPIs among the 72 SSU processome proteins (Table 1).

### A poor overlap for the HT-Y2H, PCA and LC datasets

**Figure 5:**
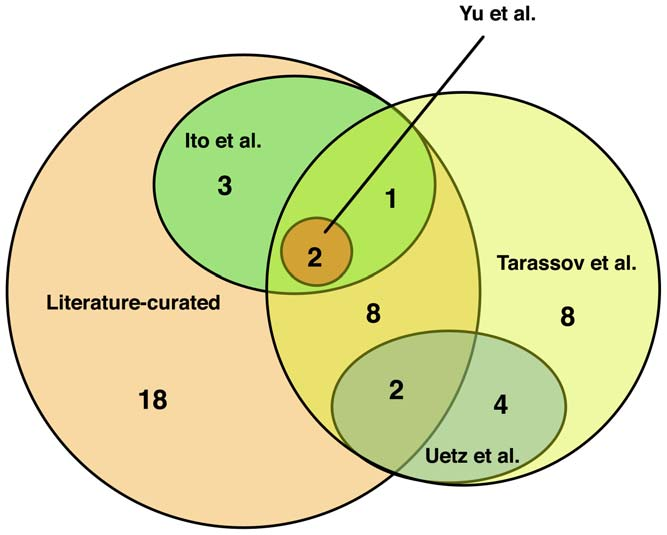
Comparison of the overlap between the HT-Y2H, PCA and LC datasets for the PPIs of the SSU processome. Numbers within the Venn diagram refer to the number of SSU processome proteins present and overlapping in the HT-Y2H, PCA and LC datasets.

Interactions identified by different studies or using independent methods carry a higher confidence value [76]–[77]. Therefore, we examined the level of overlap between the genome-wide HT-Y2H studies, the PCA and LC datasets. Minimal congruence was found among the HT-Y2H datasets, with Uetz *et al.* [1] and Ito *et al.* [2] not sharing any reported interactions (Figs. 1 and 5). The SSU processome interactions reported by Yu *et al.* [3] overlap completely with those of Ito *et al.* [2] and were thus already known. The interactions reported in the systems biology study of Hazbun *et al.* [5] do not overlap with any of the HT-Y2H datasets [1]–[3]. Some overlap was found between the HT-Y2H studies [1]–[3] and the PCA dataset [4] (nine proteins and four PPIs; Figs. 1, 2 and 5). Overlap was also found between the HT-Y2H studies [1]–[3], the PCA dataset [4] and the LC dataset (Figs. 1, 2, 3 and 5) [37]–[51]. However, 18 of the 34 proteins in the LC dataset did not overlap with any of the HT-Y2H [1]–[3] or PCA [4] studies.

## Discussion

Large-scale, genome-wide yeast binary protein interaction networks contain thousands of PPIs suggesting comprehensive and complete investigations of the yeast interactome. We mined the existing databases, containing PPIs from all HT-Y2H [1]–[3], [5], PCA [4] and LC [37]–[51] yeast interactome studies to date for interactions among the 72 SSU processome proteins. Individual datasets were analyzed for the extent of PPI coverage and overlap and were merged to generate one comprehensive interaction dataset. Individual datasets and their amalgamation were each drawn into interactome maps using Cytoscape. Our results show that filtering the current HT-Y2H [1]–[3], [5], PCA [4] and LC [37]–[51] datasets for SSU processome PPIs provided sparse data, with as many as 36.1% (26 of 72 SSU processome proteins) of the protein components having no currently known interaction partner. A strategy similar to ours has successfully been used to draw an interaction map of promyelocytic leukaemia protein nuclear bodies (PML-NBs) [78].

### How many protein-protein interactions are expected?

There are approximately 6,000 proteins and a conservative estimate of 18,000+/−4500 PPIs in the entire yeast interactome [3], [79]–[81], equaling an average of 3 to 3.5 interactions per protein (though this number may be as high as five interactions per protein [82]). By this calculation, for 72 SSU processome proteins, we expected roughly 216 to 252 PPIs in total (Table 1). Based on the lower end of the theoretical number of expected PPIs, the 67 PPIs that we obtained from the merged datasets represent at most 31.0% of the predicted interactions in the SSU processome (Table 1). This number is in line with similar estimates from merged HT-Y2H datasets suggesting ∼20% coverage of the entire yeast interactome [3]. From these values, it is clear that we do not yet have an interactome of the SSU processome that is nearly complete.

### Comparing the HT-Y2H, PCA and LC datasets

Among the genome-wide studies, the PCA dataset of Tarassov *et al.* [4] reports the highest PPI coverage when compared to the three HT-Y2H-based approaches [1]–[3], accounting for 25 SSU processome proteins and 12.5 percent of the predicted edges (Table 1). This might be attributed to the distinctiveness of the PCA method [83] and to the screening strategy, which involved a one-by-one matrix array where each bait-containing strain was individually mated to each prey-containing strain [4]. In contrast, the prey pooling approach used in the Uetz *et al.* [1], Ito *et al.* [2] and Yu *et al.* [3] HT-Y2H studies has potentially lower quality data and coverage, possibly because: *i*) some prey plasmids may replicate faster due to their smaller size, and can overtake the population in the pool by outcompeting larger prey plasmids that take longer or are more difficult to replicate, *ii*) some proteins, when over-expressed, may be toxic to the cell resulting in a dominant negative phenotype, while other proteins can enhance cell growth (cells with improved growth can outcompete other cells, while those with a dominant negative phenotype will be eliminated from the pool) and *iii*) there may be transformation and mating differences among different prey fusion protein plasmids [35], [52]. Furthermore, array-based screened may be more sensitive and more easily screened to saturation [35], [52]. Thus, the individualized mating process used by Tarassov *et al.* [4], which avoids many of the potential problems associated with the pooling approach, could explain their higher coverage of the SSU processome protein interactome.

Protein interactions reported by more than one study, replicated via distinct methods or reported in different organisms are more likely to be authentic [76]–[77]. As has been found in other studies [4], [36], [83]–[85], inspection and comparison among the compiled HT-Y2H, PCA and LC datasets, however, revealed poor overlap, especially among the genome-wide HT-Y2H datasets [1]–[3] which contained very few overlapping PPIs. Due to the large contributions of the LC [37]–[51] and PCA [4] datasets to the interaction map of the SSU processome, most of the overlaps occurred between the LC and PCA datasets (Figs. 2, 3 and 5). The poor overlap among the comprehensive HT-Y2H interactomes brings into question their proposed completeness and suggests that these screens were not exhaustive nor done to saturation.

### The high quality of the LC dataset

Smaller-scale LC datasets provided the highest coverage of the SSU processome proteins, reporting 34 proteins and 44 interactions (47.2% and 20.4% of the predicted totals, respectively). While conventional wisdom supports LC datasets to be accurate and of high-quality, some have remained skeptical, pointing to the poor overlap among the literature-curated studies, as well as protein name and species classification errors [19], [21], [31]–[32]. Surveys to assess the reliability of literature-curated data by re-curation revealed roughly half of LC derived data to lack validation via alternative, independent methods [19], [21], [31]. In contrast to these claims, our analysis revealed the LC data to be the most comprehensive. Furthermore, many of the SSU processome PPIs from the mined LC dataset were found to be validated by independent methods such as *E. coli* pull-downs and biochemical and biophysical assays (Table 2).

**Table 2:** Y2H-derived PPI data confirmed by alternative and supplementary experimental methods.

Interactor A	Interactor B	Y2H	GST/His-Tag pull-down	Biochemical activation	Motif mapping	Surface plasmon resonance
Imp3	Mpp10	**✓** [46]	**✓** [46]		**✓** [46]	
Imp4	Mpp10	**✓** [46]	**✓** [46]		**✓** [46]	
Esf2	Dbp8	**✓** [43]	**✓** [43]	**✓** [43]		
Pfa1	Prp43	**✓** [48], [89]	**✓** [89]	**✓** [89]	**✓** [89]	
t-Utp8	t-Utp9	**✓** [39], [44]	**✓** [44]	**✓** [44]	**✓** [44]	
Utp6	Utp21	**✓** [38]			**✓** [38]	**✓** [38]
Utp6	Utp18	**✓** [38]			**✓** [38]	
Utp25	Utp3	**✓** [41], [51]	**✓** [41]		**✓** [41], [51]	

Many PPIs from the LC data have alternative forms of supporting evidence from experiments that test for binary interactions, including pull-downs, activation of enzymatic activities, motif mapping by truncations and surface plasmon resonance. This list of protein-protein interactions identified by Y2H and validated by independent methods is not exhaustive.

### Sparse interologue data for SSU processome components

The use of interologues in protein-protein interaction maps is rapidly increasing and constitutes a valid strategy for augmenting interactome coverage [77]. Some of the PPIs identified by multiple studies, such as between Imp3 and Mpp10, and Imp4 and Mpp10, were also reported in different organisms such as *Drosophila* [6]. Although all 72 SSU processome components were queried in six additional organisms other than *S. cerevisiae*, the majority of retrieved PPIs were with non-SSU processome proteins or with proteins with no known yeast orthologues. Once the SSU processome components of various model organisms are better characterized, and their yeast orthologues determined, additional conserved interactions may be identified. However, our analysis suggests that the interactome coverage of *C. elegans*, *D. melanogaster*, *S. pombe*, *P. falciparum*, human and mouse may be even less than that of yeast. This is in line with a recent report suggesting that low interactome coverage, and not evolutionary divergence and loss of interologues, as the main obstacle to interactome network alignment [86].

### What does this tell us about the SSU processome protein-protein interaction map?

A few novel interactions previously undetected by HT-Y2H and LC studies surfaced in the PCA dataset: between t-Utp4 and t-Utp10, t-Utp5 and t-Utp8, t-Utp5 and t-Utp9, and t-Utp8 and t-Utp15 of the UtpA/t-Utp subcomplex and between Utp1 and Utp12 of the UtpB subcomplex (compare Figs. 2 and 3). The identification of these interactions in the PCA dataset [4] but not in the HT-Y2H or LC datasets [38]–[39] may be due to differences between the Y2H and PCA methodologies [83] or to differences resulting from the use of different fusion tags in Y2H and PCA screening strategies. Indeed, the N- versus C-terminal placement of fusion tags in Y2H assays has been shown to influence the outcome of screens [87]. Regardless, validating these PCA derived interactions will further clarify the assembly of the t-Utp/UtpA and UtpB subcomplexes of the SSU processome.

Novel interactions were also reported between t-Utp4 of the UtpA/t-Utp and Utp18 of the UtpB subcomplexes. This interaction may suggest one of the first PPIs linking the various subcomplexes of the SSU processome, and is also a candidate for future validation studies. Interestingly, all genome-wide HT-Y2H screens [1]–[3] are missing these interactions, potentially due to these findings being either an artifact of the PCA approach, or a false negative of the Y2H methodology. False negatives in Y2H screens may arise from bait and prey proteins that normally interact via their N-terminus, since the DNA binding or activation domains, which are typically attached to the N-terminus of the proteins, may mask these interaction surfaces.

A truly comprehensive interactome map of the SSU processome will provide us with insight into the complexities of the assembly, function and regulation of this large ribonucleoprotein complex. Since the SSU processome is required for the production of ribosomes in all eukaryotes, understanding its assembly is essential to elucidating its function in ribosome biogenesis. Our analyses of the existing databases indicates that ∼70% of the PPIs in the SSU processome have yet to be determined, and because of this we do not yet have an accurate picture of how this complex is assembled. The current lack of data includes both proteins with no known interactors, and missing PPIs between other connected proteins. Enhancing the experimental approaches to both the classic methods — such as the Y2H — and new methods — such as the PCA — are likely to be crucial for not only deriving an interactome map of the SSU processome, but a comprehensive and exhaustively screened yeast PPI map that covers the entire yeast proteome.

This quantitative survey of existing databases for PPIs from HT-Y2H [1]–[3], PCA [4] and LC [37]–[51] studies reveals a remarkably sparse coverage of the SSU processome proteins, albeit having drawn data from interactomes purporting to be highly comprehensive. Nevertheless, the absence of a truly comprehensive, genome-wide interactome is apparent.

The LC dataset, which provided the highest coverage of the SSU processome proteins, contained PPIs that were confirmed by alternative methods, such as *E. coli* pull-downs and biochemical and biophysical methods that also test for direct binary interactions. This confirms that PPIs from LC sources, despite previously proposed skepticism, are largely credible.

Although lacking many proteins and interactions, the up-to-date SSU processome interaction map compiled in this study can be applied to generate new hypotheses of subcomplex interactions, assembly and function. Additionally, approaches to experimentally determine the domain-domain interactions of the known PPIs [88] can be applied to better understand the biology of the SSU processome.

## Supporting Information

Table S1 **The protein components of the SSU processome.** The catalogued proteins are listed based on their membership in the known subcomplexes of the yeast SSU processome. Confirmed SSU processome components which have not been assigned to a specific subcomplex are listed as unclassified. Candidate SSU processome proteins are listed as unknown. The yeast SSU r-proteins (Rps4, Rps6, Rps7, Rps9 and Rps14) that are known components of the SSU processome [54] are not listed. (?) denotes uncertain membership in an SSU processome sub-complex. Motif and domain abbreviations include: glycine/arginine-rich (GAR); coiled-coil (CC); middle domain of eIF4G (MIF4G); MA3 domain (similar to MIF4G domains/MI domain); helicase conserved C-terminal domain (HELICc); helicase associated domain (HA2); glycine-rich nucleic binding domain (G-patch); RxxxH ssRNA binding motif (R3H); Pumilio homology RNA binding domain (PUM/PUF); RNA recognition motif (RRM, RBD or RNP domain); low-temperature viability protein domain (LTV1); fungal-specific family of rRNA processing proteins (rRNA processing domain); small domain in a novel nucleolar family (NUC153); beta-transducin repeats (WD40); S1 RNA-binding motifs; Half-A-TPR (HAT) repeats; K homology RNA-binding domain (KH); Down-Regulated In Metastasis (DRIM); Armadillo (ARM) protein-protein interaction repeat; CBF/Mak21 family; nucleolar complex (NOC) associated protein domain. Table modified from Phipps *et al.* [55].
(DOC)

Table S2 **The SSU processome PPIs derived from the HT-Y2H, PCA and LC datasets.**
(XLS)
