# Supplementary material for: Assembling a Protein-Protein Interaction Map of the SSU Processome from Existing Datasets
Source: PLoS One. 2011 Mar 10;6(3):e17701. doi: 10.1371/journal.pone.0017701 (PMC3053386; doi:10.1371/journal.pone.0017701)
Supplement: Table S1 — The protein components of the SSU processome. The catalogued proteins are listed based on their membership in the known subcomplexes of the yeast SSU processome. Confirmed SSU processome components which have not been assigned to a specific subcomplex are listed as unclassified. Candidate SSU processome proteins are listed as unknown. The yeast SSU r-proteins (Rps4, Rps6, Rps7, Rps9 and Rps14) that are known components of the SSU processome [54] are not listed. (?) denotes uncertain membership in an SSU processome sub-complex. Motif and domain abbreviations include: glycine/arginine-rich (GAR); coiled-coil (CC); middle domain of eIF4G (MIF4G); MA3 domain (similar to MIF4G domains/MI domain); helicase conserved C-terminal domain (HELICc); helicase associated domain (HA2); glycine-rich nucleic binding domain (G-patch); RxxxH ssRNA binding motif (R3H); Pumilio homology RNA binding domain (PUM/PUF); RNA recognition motif (RRM, RBD or RNP domain); low-temperature viability protein domain (LTV1); fungal-specific family of rRNA processing proteins (rRNA processing domain); small domain in a novel nucleolar family (NUC153); beta-transducin repeats (WD40); S1 RNA-binding motifs; Half-A-TPR (HAT) repeats; K homology RNA-binding domain (KH); Down-Regulated In Metastasis (DRIM); Armadillo (ARM) protein-protein interaction repeat; CBF/Mak21 family; nucleolar complex (NOC) associated protein domain. Table modified from Phipps et al. [55]. (DOC) [file pone.0017701.s001.doc]

| Name | Alias | **Sub-Complex** | Comments | Motifs/Domains | References |
| --- | --- | --- | --- | --- | --- |
| Nop1 | Fib/Lot3 | Box C/D | 2'-*O*-methyltransferase | N-term GAR domain; C-term fibrillarin domain | [1-2] |
| Nop56 | Sik1 | Box C/D | KKE/D repeats | N-term NOP5NT domain; central NOSIC and NOP domains;  C-term CC and RNA Pol I Site | [3] |
| Nop58 | Nop5 | Box C/D | KKE/D repeats | N-term NOP5NT domain; central NOSIC domain;  C-term CC and RNA Pol I Site | [4] |
| Snu13 |  | Box C/D | binds to the helix-bulge-helix (K-turn) motifs of U3 snoRNA; also part of the U4/U6-U5 tri-snRNP | N-term ribosomal L7Ae domain | [5] |
| Rrp9 |  | U3 snoRNP | binds to the B/C motif of U3 snoRNA | N-term CC; five WD40 repeat motifs | [6-7] |
| Imp3 |  | Mpp10 |  | C-term S4 RBD | [8] |
| Imp4 |  | Mpp10 | contains a σ^70^-like motif | N-term CC; C-term BRIX domain | [8] |
| Mpp10 |  | Mpp10 | associated with the hinge region of U3 snoRNA | central CC | [9] |
| t-Utp4 |  | UtpA |  | three N-term WD40 repeats | [10-12] |
| t-Utp5 |  | UtpA |  | N-term WD40 repeat; C-term CC | [10-11] |
| t-Utp8 |  | UtpA | also involved in nuclear tRNA export | Low-complexity regions; no known motifs or domains | [10-11,13] |
| t-Utp9 |  | UtpA | WD40 repeats | Low-complexity regions; no known motifs or domains | [10-12] |
| t-Utp10 |  | UtpA |  | C-term BP28CT (NUC211) domain and HEAT repeats | [10-12] |
| t-Utp15 |  | UtpA |  | Six N-term WD40 repeats | [10-12] |
| t-Utp17 | Nan1 | UtpA | also part of RENT complex | three WD40 repeats | [10-12] |
| Pol5 |  | UtpA | required for rRNA synthesis; not required for DNA replication | N-terminal CC | [11-12,14] |
| Utp1 | Pwp2 | UtpB | β transducin family | WD 40 repeats, C-term Pwp2 domain | [10,12,15-16] |
| Utp6 |  | UtpB | half-a-tetratricopeptide repeat motif [half-a-TPR (HAT)] | N-term HAT motifs | [10,12,15-16] |
| Utp12 | Dip2 | UtpB |  | C-term Dip2 domain; central and C-term CC, WD40 repeats | [10,12,15-16] |
| Utp13 |  | UtpB |  | C-term CC and Utp13 domain | [10,12,15-16] |
| Utp18 |  | UtpB |  | WD40 repeats | [12,15-17] |
| Utp21 |  | UtpB |  | WD40 repeats, C-terminal Utp21 domain | [12,15-18] |
| Rrp7 |  | UtpC | Also part of the CURI complex | Low-complexity regions; no known motifs or domains | [12,19-20] |
| Utp22 |  | UtpC | Also part of the CURI complex | C-term NRAP domain | [12,17-18,20-21] |
| Cka1 |  | UtpC | Co-purifies with UtpC; α catalytic subunit of casein kinase 2; also part of the CURI complex; non-essential | central STYKc domain | [12,20] |
| Cka2 |  | UtpC | Co-purifies with UtpC; α’ catalytic subunit of casein kinase 2; also part of the CURI complex; non-essential | central STYKc domain | [12,20] |
| Ckb1 |  | UtpC | Co-purifies with UtpC; β regulatory subunit of casein kinase 2; also part of the CURI complex; non-essential | central STYKc domain | [12,20] |
| Ckb2 |  | UtpC | Co-purifies with UtpC; β’ regulatory subunit of casein kinase 2; also part of the CURI complex; non-essential | central Ck2β domain | [12,20] |
| Rrp36 |  | UtpC? |  | C-term CC and DUF947 domain | [22] |
| Bms1 |  | Bms1/Rcl1 | GTPase, stimulated by Rcl1 | N-term GTP EFTu BD & AARP2CN domain; C-term DUF663 domain & CC | [23-24] |
| Rcl1 |  | Bms1/Rcl1 | RNA terminal phosphate cyclase-like protein;  stimulates Bms1 | RTC and RTC insert domains | [25] |
| Utp2 | Nop14 | unclassified | also involved in SSU nuclear export | Multiple CC; Nop14 domain | [10,26-27] |
| Utp3 | Sas10 | unclassified | also involved in gene silencing | Mutiple CC (N and C-term); central and C-term Utp3 domain | [10] |
| Utp7 | Kre31 | unclassified | adenylate binding site; kinetochore component  required for chromosome segregation | WD40 repeats; C-term BING4CT domain | [10,28] |
| Utp11 |  | unclassified |  | C-term CC | [10] |
| Utp14 |  | unclassified |  | central CC; central and C-term Utp14 domain | [10] |
| Utp16 | Bud21 | unclassified | non-essential | U3 snoRNA associated superfamily domain | [10] |
| Noc4 | Utp19 | unclassified | also involved in SSU nuclear export | C-term CBF/Mak21 and NOC domains | [17,27] |
| Utp20 |  | unclassified | 11 ARM repeats | central DRIM domain, 2 C-term CCs | [17-18] |
| Utp23 |  | unclassified | PINc nuclease domain not required for function;  probable paralogue of Utp24 | central PINc domain | [29] |
| Utp24 | Fcf1 | unclassified | putative SSU processome endonuclease; PINc nuclease domain required for function | central and C-term PINc domain | [29] |
| Utp25 |  | unclassified | DEAD-box helicase-like | central and C-term DUF1253 domain | [30-32] |
| Utp30 |  | unclassified | non-essential | Member of the ribosomal protein L1 superfamily | [18] |
| Dbp8 |  | unclassified | DEAD-box helicase; stimulated by Esf2 | N-term DEAD-box domain; central CC and HELICc domain | [33] |
| Dhr1 | Ecm16 | unclassified | DEAH-box helicase | N-term low complexity; central DEAH and HELICc domains;  C-term HA2 and DUF1605 domains | [33-34] |
| Dhr2 |  | unclassified | DEAH-box helicase | N-term DEAD-box; central HELICc and HA2; C-term DUF1605 domain | [33-34] |
| Emg1 | Nep1 | unclassified | member of α/β knot fold methyltransferase (SPOUT) superfamily; interacts with RNA and with SAM; displays pseudouridine methyltransferase activity | central and C-term Emg1 domain | [17,35] |
| Krr1 |  | unclassified | contains a KRR-R motif and a KH domain | N-term KRR-R motif; central KH domain; C-term CC | [17,36] |
| Rok1 |  | unclassified | DEAD-box helicase | central DEAD-box and HELICc domains | [33] |
| Rrp3 |  | unclassified | DEAD-box helicase | N-term CC; central DEAD; C-term HELICc domain | [33] |
| Rrp5 |  | unclassified | binds single stranded tracts of U's; also participates  in A3 cleavage in 5.8S rRNA processing | 12 N-term and central S1 motifs; C-term CC and 6 HAT motifs | [10,37] |
| Sof1 |  | unclassified | similar to β subunit of trimeric G-proteins | 2 N-term WD40 repeats; 2 central WD40s; C-term WD40 and Sof1 domain | [38] |
| Dbp4 | Hca4/Ecm24 | unknown | DEAD-box helicase | N-term DEAD box; central HELICc motif; C-terminal CC | [39] |
| Enp1 | Meg1 | unknown | contains a Bystin domain | N-term KKE/D; central Bystin and CC domains | [17,40] |
| Esf1 |  | unknown |  | N-term low complexity; central CC; C-term NUC153 motif and KKE/D repeats | [41] |
| Esf2 | Abt1 | unknown | binds to RNA and stimulates ATPase activity of Dbp8 | N-term CC; central RRM; C-term CC | [42-43] |
| Fal1 |  | unknown | member of eIF4A subfamily of DEAD-box helicases | central DEAD domain; C-term HELICc domain | [44] |
| Fyv7 |  | unknown | contains coiled-coil motifs; non-essential | rRNA processing domain; central CC and KKE/D motifs | [21] |
| Gno1 | Pxr1 | unknown | also involved in snoRNA maturation | N-term G-patch; central KKE/D and CC | [45] |
| Has1 |  | unknown | DEAD-box helicase; also required for LSU synthesis | N-term DEAD motif; central HELICc domain | [46] |
| Kre33 |  | unknown | putative N-acetyltransferase and ATPase domains | N-term DUF1726; central DUF699 (putative ATPase); C-term CC | [47-48] |
| Lcp5 |  | unknown | contains a Utp3 domain | N-term Utp3 domain and CC; central and C-term low complexity regions | [49] |
| Ltv1 | Ykl2 | unknown | also required for SSU nuclear export; non-essential | LTV1 domain; central CC; C-terminal CC | [50] |
| Mrd1 |  | unknown | contains 5 RNA-binding domains | N-term RRM and CC; 2 central RRMs; C-term CC and 2 RRMs | [51] |
| Nop9 |  | unknown | multiple pumilio-like RNA binding repeats | 3 N-term PUMs; 3 central PUMs; 2 C-term PUMs | [52] |
| Nsr1 | She5 | unknown |  | Central RRM and CC; C-term RRM and GAR motifs | [53-54] |
| Pfa1 | Sqs1 | unknown | stimulates Prp43; non-essential | C-term R3H and G-patch domains | [55] |
| Prp43 | JA1 | unknown | DEAH-box helicase; also involved in mRNA splicing  and LSU biosynthesis | N-terminal helicase; central HELICc and HA2 domains;  C-term DUF1605 and 2 CCs | [56] |
| Sen1 | Cik3/Nrd2 | unknown | Upf1-like helicase; also required for LSU synthesis | N-term extension; central helicase and 2 CCs; C-term CC and extension | [57] |
| Sgd1 |  | unknown | contains armadillo-type fold | N-term CC; central MIF4G; C-term MA3 domains | [48] |
| Slx9 |  | unknown | non-essential | Low-complexity regions; no known motifs or domains | [58] |
| YGR251W |  | unknown |  | N-term DUF2702 | [21] |

References

1. Tollervey D, Lehtonen H, Carmo-Fonseca M, Hurt EC (1991) The small nucleolar RNP protein NOP1 (fibrillarin) is required for pre-rRNA processing in yeast. EMBO J 10: 573-583.

2. Lischwe MA, Ochs RL, Reddy R, Cook RG, Yeoman LC, et al. (1985) Purification and partial characterization of a nucleolar scleroderma antigen (Mr = 34,000; pI, 8.5) rich in NG,NG-dimethylarginine. J Biol Chem 260: 14304-14310.

3. Lafontaine DL, Tollervey D (2000) Synthesis and assembly of the box C+D small nucleolar RNPs. Mol Cell Biol 20: 2650-2659.

4. Wu P, Brockenbrough JS, Metcalfe AC, Chen S, Aris JP (1998) Nop5p is a small nucleolar ribonucleoprotein component required for pre-18 S rRNA processing in yeast. J Biol Chem 273: 16453-16463.

5. Watkins NJ, Segault V, Charpentier B, Nottrott S, Fabrizio P, et al. (2000) A common core RNP structure shared between the small nucleoar box C/D RNPs and the spliceosomal U4 snRNP. Cell 103: 457-466.

6. Venema J, Vos HR, Faber AW, van Venrooij WJ, Raue HA (2000) Yeast Rrp9p is an evolutionarily conserved U3 snoRNP protein essential for early pre-rRNA processing cleavages and requires box C for its association. RNA 6: 1660-1671.

7. Lukowiak AA, Granneman S, Mattox SA, Speckmann WA, Jones K, et al. (2000) Interaction of the U3-55k protein with U3 snoRNA is mediated by the box B/C motif of U3 and the WD repeats of U3-55k. Nucleic Acids Res 28: 3462-3471.

8. Lee SJ, Baserga SJ (1999) Imp3p and Imp4p, two specific components of the U3 small nucleolar ribonucleoprotein that are essential for pre-18S rRNA processing. Mol Cell Biol 19: 5441-5452.

9. Dunbar DA, Wormsley S, Agentis TM, Baserga SJ (1997) Mpp10p, a U3 small nucleolar ribonucleoprotein component required for pre-18S rRNA processing in yeast. Mol Cell Biol 17: 5803-5812.

10. Dragon F, Gallagher JE, Compagnone-Post PA, Mitchell BM, Porwancher KA, et al. (2002) A large nucleolar U3 ribonucleoprotein required for 18S ribosomal RNA biogenesis. Nature 417: 967-970.

11. Gallagher JE, Dunbar DA, Granneman S, Mitchell BM, Osheim Y, et al. (2004) RNA polymerase I transcription and pre-rRNA processing are linked by specific SSU processome components. Genes Dev 18: 2506-2517.

12. Krogan NJ, Peng WT, Cagney G, Robinson MD, Haw R, et al. (2004) High-definition macromolecular composition of yeast RNA-processing complexes. Mol Cell 13: 225-239.

13. Strub BR, Eswara MB, Pierce JB, Mangroo D (2007) Utp8p is a nucleolar tRNA-binding protein that forms a complex with components of the nuclear tRNA export machinery in *Saccharomyces cerevisiae*. Mol Biol Cell 18: 3845-3859.

14. Shimizu K, Kawasaki Y, Hiraga S, Tawaramoto M, Nakashima N, et al. (2002) The fifth essential DNA polymerase phi in *Saccharomyces cerevisiae* is localized to the nucleolus and plays an important role in synthesis of rRNA. Proc Natl Acad Sci U S A 99: 9133-9138.

15. Champion EA, Lane BH, Jackrel ME, Regan L, Baserga SJ (2008) A direct interaction between the Utp6 half-a-tetratricopeptide repeat domain and a specific peptide in Utp21 is essential for efficient pre-rRNA processing. Mol Cell Biol 28: 6547-6556.

16. Dosil M, Bustelo XR (2004) Functional characterization of Pwp2, a WD family protein essential for the assembly of the 90 S pre-ribosomal particle. J Biol Chem 279: 37385-37397.

17. Bernstein KA, Gallagher JE, Mitchell BM, Granneman S, Baserga SJ (2004) The small-subunit processome is a ribosome assembly intermediate. Eukaryot Cell 3: 1619-1626.

18. Samanta MP, Liang S (2003) Predicting protein functions from redundancies in large-scale protein interaction networks. Proc Natl Acad Sci U S A 100: 12579-12583.

19. Baudin-Baillieu A, Tollervey D, Cullin C, Lacroute F (1997) Functional analysis of Rrp7p, an essential yeast protein involved in pre-rRNA processing and ribosome assembly. Mol Cell Biol 17: 5023-5032.

20. Rudra D, Mallick J, Zhao Y, Warner JR (2007) Potential interface between ribosomal protein production and pre-rRNA processing. Mol Cell Biol 27: 4815-4824.

21. Peng WT, Robinson MD, Mnaimneh S, Krogan NJ, Cagney G, et al. (2003) A panoramic view of yeast noncoding RNA processing. Cell 113: 919-933.

22. Gérus M, Bonnart C, Caizergues-Ferrer M, Henry Y, Henras AK (2010) Evolutionarily conserved function of RRP36 in early cleavages of the pre-rRNA and production of the 40S ribosomal subunit. Mol Cell Biol 30: 1130-1144.

23. Karbstein K, Jonas S, Doudna JA (2005) An essential GTPase promotes assembly of preribosomal RNA processing complexes. Mol Cell 20: 633-643.

24. Wegierski T, Billy E, Nasr F, Filipowicz W (2001) Bms1p, a G-domain-containing protein, associates with Rcl1p and is required for 18S rRNA biogenesis in yeast. RNA 7: 1254-1267.

25. Billy E, Wegierski T, Nasr F, Filipowicz W (2000) Rcl1p, the yeast protein similar to the RNA 3'-phosphate cyclase, associates with U3 snoRNP and is required for 18S rRNA biogenesis. EMBO J 19: 2115-2126.

26. Liu PC, Thiele DJ (2001) Novel stress-responsive genes EMG1 and NOP14 encode conserved, interacting proteins required for 40S ribosome biogenesis. Mol Biol Cell 12: 3644-3657.

27. Milkereit P, Strauss D, Bassler J, Gadal O, Kuhn H, et al. (2003) A Noc complex specifically involved in the formation and nuclear export of ribosomal 40 S subunits. J Biol Chem 278: 4072-4081.

28. Jwa M, Kim JH, Chan CS (2008) Regulation of Sli15/INCENP, kinetochore, and Cdc14 phosphatase functions by the ribosome biogenesis protein Utp7. J Cell Biol 182: 1099-1111.

29. Bleichert F, Granneman S, Osheim YN, Beyer AL, Baserga SJ (2006) The PINc domain protein Utp24, a putative nuclease, is required for the early cleavage steps in 18S rRNA maturation. Proc Natl Acad Sci USA 103: 9464-9469.

30. Goldfeder MB, Oliveira CC (2010) Utp25p, a nucleolar *Saccharomyces cerevisiae* protein, interacts with U3 snoRNP subunits and affects processing of the 35S pre-rRNA. FEBS J 277: 2838-2852.

31. Charette JM, Baserga SJ (2010) The DEAD-box RNA helicase-like Utp25 is an SSU processome component. RNA 16: 2156-2169.

32. Harscoët E, Dubreucq B, Palauqui JC, Lepiniec L (2010) NOF1 encodes an arabidopsis protein involved in the control of rRNA expression. PLoS ONE 5: e12829.

33. Granneman S, Bernstein KA, Bleichert F, Baserga SJ (2006) Comprehensive mutational analysis of yeast DEXD/H box RNA helicases required for small ribosomal subunit synthesis. Mol Cell Biol 26: 1183-1194.

34. Colley A, Beggs JD, Tollervey D, Lafontaine DL (2000) Dhr1p, a putative DEAH-box RNA helicase, is associated with the box C+D snoRNP U3. Mol Cell Biol 20: 7238-7246.

35. Wurm JP, Meyer B, Bahr U, Held M, Frolow O, et al. (2010) The ribosome assembly factor Nep1 responsible for Bowen-Conradi syndrome is a pseudouridine-N1-specific methyltransferase. Nucleic Acids Res 38: 2387-2398.

36. Gromadka R, Kaniak A, Slonimski PP, Rytka J (1996) A novel cross-phylum family of proteins comprises a KRR1 (YCL059c) gene which is essential for viability of *Saccharomyces cerevisiae* cells. Gene 171: 27-32.

37. Venema J, Tollervey D (1996) RRP5 is required for formation of both 18S and 5.8S rRNA in yeast. EMBO J 15: 5701-5714.

38. Jansen R, Tollervey D, Hurt EC (1993) A U3 snoRNP protein with homology to splicing factor PRP4 and G beta domains is required for ribosomal RNA processing. EMBO J 12: 2549-2558.

39. Liang WQ, Clark JA, Fournier MJ (1997) The rRNA-processing function of the yeast U14 small nucleolar RNA can be rescued by a conserved RNA helicase-like protein. Mol Cell Biol 17: 4124-4132.

40. Chen W, Bucaria J, Band DA, Sutton A, Sternglanz R (2003) Enp1, a yeast protein associated with U3 and U14 snoRNAs, is required for pre-rRNA processing and 40S subunit synthesis. Nucleic Acids Res 31: 690-699.

41. Peng WT, Krogan NJ, Richards DP, Greenblatt JF, Hughes TR (2004) ESF1 is required for 18S rRNA synthesis in *Saccharomyces cerevisiae*. Nucleic Acids Res 32: 1993-1999.

42. Hoang T, Peng WT, Vanrobays E, Krogan N, Hiley S, et al. (2005) Esf2p, a U3-associated factor required for small-subunit processome assembly and compaction. Mol Cell Biol 25: 5523-5534.

43. Granneman S, Lin C, Champion EA, Nandineni MR, Zorca C, et al. (2006) The nucleolar protein Esf2 interacts directly with the DExD/H box RNA helicase, Dbp8, to stimulate ATP hydrolysis. Nucleic Acids Res 34: 3189-3199.

44. Kressler D, de la Cruz J, Rojo M, Linder P (1997) Fal1p is an essential DEAD-box protein involved in 40S-ribosomal-subunit biogenesis in *Saccharomyces cerevisiae*. Mol Cell Biol 17: 7283-7294.

45. Guglielmi B, Werner M (2002) The yeast homolog of human PinX1 is involved in rRNA and small nucleolar RNA maturation, not in telomere elongation inhibition. J Biol Chem 277: 35712-35719.

46. Emery B, de la Cruz J, Rocak S, Deloche O, Linder P (2004) Has1p, a member of the DEAD-box family, is required for 40S ribosomal subunit biogenesis in *Saccharomyces cerevisiae*. Mol Microbiol 52: 141-158.

47. Grandi P, Rybin V, Baßler J, Petfalski E, Strauß D, et al. (2002) 90S pre-ribosomes include the 35S pre-rRNA, the U3 snoRNP, and 40S subunit processing factors but predominantly lack 60S synthesis factors. Mol Cell 10: 105-115.

48. Li Z, Lee I, Moradi E, Hung NJ, Johnson AW, et al. (2009) Rational extension of the ribosome biogenesis pathway using network-guided genetics. PLoS Biol 7: e1000213.

49. Wiederkehr T, Prétôt RF, Minvielle-Sebastia L (1998) Synthetic lethal interactions with conditional poly(A) polymerase alleles identify LCP5, a gene involved in 18S rRNA maturation. RNA 4: 1357-1372.

50. Loar JW, Seiser RM, Sundberg AE, Sagerson HJ, Ilias N, et al. (2004) Genetic and biochemical interactions among Yar1, Ltv1 and Rps3 define novel links between environmental stress and ribosome biogenesis in *Saccharomyces cerevisiae*. Genetics 168: 1877-1889.

51. Jin SB, Zhao J, Bjork P, Schmekel K, Ljungdahl PO, et al. (2002) Mrd1p is required for processing of pre-rRNA and for maintenance of steady-state levels of 40 S ribosomal subunits in yeast. J Biol Chem 277: 18431-18439.

52. Thomson E, Rappsilber J, Tollervey D (2007) Nop9 is an RNA binding protein present in pre-40S ribosomes and required for 18S rRNA synthesis in yeast. RNA 13: 2165-2174.

53. Kondo K, Inouye M (1992) Yeast NSR1 protein that has structural similarity to mammalian nucleolin is involved in pre-rRNA processing. J Biol Chem 267: 16252-16258.

54. Lee WC, Zabetakis D, Mélèse T (1992) NSR1 is required for pre-rRNA processing and for the proper maintenance of steady-state levels of ribosomal subunits. Mol Cell Biol 12: 3865-3871.

55. Lebaron S, Papin C, Capeyrou R, Chen YL, Froment C, et al. (2009) The ATPase and helicase activities of Prp43p are stimulated by the G-patch protein Pfa1p during yeast ribosome biogenesis. EMBO J 28: 3808-3819.

56. Combs DJ, Nagel RJ, Ares M, Jr., Stevens SW (2006) Prp43p is a DEAH-box spliceosome disassembly factor essential for ribosome biogenesis. Mol Cell Biol 26: 523-534.

57. Ursic D, Himmel KL, Gurley KA, Webb F, Culbertson MR (1997) The yeast SEN1 gene is required for the processing of diverse RNA classes. Nucleic Acids Res 25: 4778-4785.

58. Bax R, Raué HA, Vos JC (2006) Slx9p facilitates efficient ITS1 processing of pre-rRNA in *Saccharomyces cerevisiae*. RNA 12: 2005-2013.
